# Novel Gold Nanoparticle-Based Quick Small-Exosome Isolation Technique from Serum Sample at a Low Centrifugal Force

**DOI:** 10.3390/nano12101660

**Published:** 2022-05-13

**Authors:** Krishna Thej Pammi Guru, Jamuna Surendran Sreeja, Dhrishya Dharmapal, Suparna Sengupta, Palash Kumar Basu

**Affiliations:** 1 Department of Avionics, Indian Institute of Space Science and Technology, Thiruvanathapuram 695547, India; krishnathej@gmail.com; 2 Rajiv Gandhi Center for Biotechnology, Thiruvananthapuram 695014, India; jssreeja@rgcb.res.in (J.S.S.); dhrishya@rgcb.res.in (D.D.)

**Keywords:** Au nanoparticle synthesis, small exosome isolation, PEG, EDC-SNHS chemistry, CD63

## Abstract

Exosomes are cell-secreted vesicles secreted by a majority of cells and, hence, populating most of the biological fluids, namely blood, tears, sweat, swab, urine, breast milk, etc. They vary vastly in size and density and are influenced by age, gender and diseases. The composition of exosomes includes lipids, DNA, proteins, and coding and noncoding RNA. There is a significant interest in selectively isolating small exosomes (≤50 nm) from human serum to investigate their role in different diseases and regeneration. However, current techniques for small exosome isolation/purification are time-consuming and highly instrument-dependent, with limited specificity and recovery. Thus, rapid and efficient methods to isolate them from bio fluids are strongly needed for both basic research and clinical applications. In the present work, we explored the application of a bench-top centrifuge for isolating mostly the small exosomes (≤50 nm). This can be achieved at low g-force by adding additional weight to the exosomes by conjugating them with citrate-capped gold nanoparticles (CGNP). CGNPs were functionalized with polyethylene glycol (PEG) to form PEGylated GNP (PGNP). EDC/SNHS chemistry is used to activate the –COOH group of the PEG to make it suitable for conjugation with antibodies corresponding to exosomal surface proteins. These antibody-conjugated PGNPs were incubated with the serum to form PGNP-exosome complexes which were separated directly by centrifugation at a low g-force of 7000× *g*. This makes this technique efficient compared to that of standard ultracentrifugation exosome isolation (which uses approximately 100,000× *g*). Using the technique, the exosome isolation from serum was achieved successfully in less than two hours. The purification of small exosomes, characterized by the presence of CD63, CD9 and CD81, and sized between 20 nm to 50 nm, was confirmed by western blot, dynamic light scattering (DLS), transmission electron microscopy (TEM) and nanoparticle tracking analyser (NTA).

## 1. Introduction

Exosomes are cell-secreted vesicles characterized by the presence of surface proteins such as CD9, CD63 and CD81. Various groups have reported diverse size ranges for exosomes. More commonly, vesicles spanning between 20–150 nm could be considered to be an optimal population of exosomes [1,2,3]. These originate as intraluminal vesicles of multivesicular bodies (MVBs) within the cell. Fusion of MVBs with the plasma membrane causes the release of these exosomes into the extracellular space. Many diverse proteins, DNA, RNA and miRNA, have been reported to be present in the lumen of the exosome [4]. Exosomes play a key role in intercellular communication [5] and are involved in the secretion of proteins for signaling pathways which in turn induce exosome secretion [6]. Exosomes have proved to be crucial in several cancers, including lung, pancreatic, liver, gastric, colorectal, renal, bladder, prostrate and ovarian cancers [7]. They also play a crucial role in the prognosis and diagnosis of cancer as they can be secreted from both healthy and tumour cells [8]. Besides cancer, exosomes also play a significant role in several diseases related to the brain, heart, immune system, Alzheimer’s, etc. [9,10].

Over the past several years, considerable progress has been made in the development of methods for the isolation of exosomes in body fluids. However, the use of exosomes and microvesicles as biomarkers to improve patient care has been limited due to their small size and the extensive sample preparation time required for their isolation. Some such widely used techniques are ultracentrifugation (UC), density gradient ultracentrifugation (DG-UC) and size exclusion chromatography (SEC). Besides this, there are several commercially available kits for the isolation of exosomes using ultrafiltration and nanoparticle-mediated separation [11,12] in which the majority of the isolated particles are in the size range of 70 nm to 150 nm. 

In the ultracentrifugation [2,13] technique, the samples are centrifuged at a speed as high as 100,000× *g*. The pellet formed after centrifugation is suspended in the desired buffer and used for processing. This is one of the widely used techniques. Multiple reports in the literature exist regarding UC with respect to speed, time of centrifugation, viscosity and volume of the starting solution. In Density Gradient Ultracentrifugation [14,15], the sample solution is placed in an ultracentrifugation tube containing inert gradient medium. Upon centrifugation, the components settled at their iso-density zone. As a result, undesired components will be co-isolated along with extracellular vesicles [16]. Similarly, size exclusion chromatography [17,18] is very popular for isolating the exosomes from biofluids. In size exclusion chromatography, the samples undergo a preliminary centrifugation at a low speed to separate the cells, cell debris, and other organelles. The supernatant from this step is passed through a porous filter whose pore diameter (0.22 µm) is sufficiently sized to isolate all the particles falling in the exosomal size range. The resulting solution is passed through a size exclusion chromatography column. Specific fractions of this column are collected for extracellular vesicle-enriched solution, which is concentrated further using centrifugal filters. In [19], authors compared the purity of exosomes isolated from UC and SEC with respect to plasma proteins (albumin). Their results confirmed the isolation of albumin in both these techniques, although SEC can be considered more efficient than UC. These methodologies (UC, DG-UC, SEC) isolate a mixture of extracellular vesicles, protein aggregates, and cell debris from a bulk solution and, hence, lack specificity as to the isolation of exosomes. Recently, magnetic separation of exosomes has become very popular. In [11], the isolation of exosomes was conducted by using magnetic beads functionalized with anti-CD9 and anti-CD81 antibodies. This technique involves preliminary low-speed centrifugation steps to separate the cell and cell debris. This is followed by incubating the supernatant with magnetic beads coated with monoclonal anti-human CD81 antibody, for 16 to 20 h at 4 °C overnight. The incubated solution was exposed to the magnetic field to pull the beads-exosome complex to the sides of the tube, followed by replacing the supernatant with PBS. This method is superior to the UC, DG-UC, and SEC techniques in terms of specificity. In [12], the authors presented a comparative study of three commercially available kits, namely TEIR, ExoQuick, and miRCURY. In brief, the serum was spun at 10,000× *g* for 10 m to remove cell debris, then mixed with the reagents as per manufacturer’s instructions and incubated at 4 °C for 1 h, followed by centrifugation (TEIR:10,000× *g*; ExoQuick and miRCURY: 1500× *g*) for 30 m to precipitate the exosome pellet. The formed pellet was dissolved in manufacturer-supplied resuspension buffer. For 250 µL of serum, approximately $10^{9}$ particles were isolated and for all the kits the mean diameter was found to be 120 ± 3 nm. The authors mentioned that contaminants such as lipoproteins, aggregated proteins, cell debris, etc. are to be expected in all the kits involving the precipitation technique. The advantages and disadvantages of these techniques are presented in Table 1. 

In [20], the authors mentioned several methods for functionalizing the gold nanoparticles. The synthesized particles were used in isolation of exosomes, but all the mentioned isolation techniques required a pre-enrichment using ultracentrifugation and no quick and comparatively cheap method of isolation of exosomes was available.

It has been observed that there is a growing interest in exosomes of different size ranges [21,22,23,24,25]. In [22], the authors performed a detailed study of particles within the exosomal size range and classified them into three groups, namely exomeres, small exosomes, and large exosomes. Upon detailed analysis, the authors found that there is a significant difference in the components associated with each of these groups. Based on the components, it is believed that the origin of these particles is also different. The particles with a size less than 50 nm are termed as exomeres, and it was reported that they lack the external membrane structure. Based on the high exomere uptake by the liver, it is also speculated that exomeres might play a significant role in metabolism during the stages of cancer progression, since the protein cargo of exomeres are significantly relevant to metabolism.

In [24], the authors attempted to study the role of small extracellular vesicles in cell culture and found that they enhance either exosome secretion or cell proliferation but not both. Upon studying the results of the cell culture supernatant treated with the exosome secretion inhibitors, namely BFA and GW4869, the authors also believed the origin of different particles in the size range 20–150 nm to be different. 

Another group [25] discovered particles with a size range comparable to exomeres but different with respect to structure and morphology. These particles were termed supermeres. Both exomeres and supermeres were isolated from cell culture media by ultracentrifugation. A detailed proteomic study was performed on all these particles and the results showed the presence of unique proteins in supermeres, exomeres, and exosomes. The authors showed that supermeres are well enriched in RNA and also concluded that the majority of the RNA that was assumed to be from exosomes was from supermeres. Enzymes relevant to glycolysis and fatty acid metabolism are enriched more in supermeres compared to those of exomeres and exosomes. Functionally, supermeres were found to alter the liver metabolism and also increase lactate secretion by the recipient cells. It was also noticed that the uptake of supermeres by the brain was high, compared to exomeres or bigger exosomes, showing that supermeres can easily cross the blood-brain barrier. The authors also showed that exomeres and supermeres have distinct effects on hepatic glucose and lipid metabolism.

Within the conventional exosomal size range, a special range to be considered is particles of size less than 50 nm. In this report, there is no definite distinction for the particles under 50 nm in terms of exosomal behaviour. Thus, we opt to refer them as the small exosomes throughout this report. Based on our survey, there is no kit available to specifically isolate small exosomes from human serum. A cost-effective, efficient method is essential for the separation and study of these small exosomes from human serum to understand more about their role in different diseases.

Three popular techniques (Turkevich–Frens, Brust–Schiffrin, and some seed growth methods) are available for in situ growth of Au nanoparticles [26]. However, all these methods involve reduction and stabilization procedures for nanoparticle synthesis. The Brust–Schiffrin technique can be used to prepare CGNPs of size less than 10 nm. The Turkevich–Frens technique can be used to synthesize 5 nm to 150 nm-sized CGNPs. However, using this technique for synthesizing particles bigger than 20 nm results in polydispersed particles. Seed growth methods are efficient in the synthesis of particles greater than 100 nm. Since smaller CGNPs prepared by the Brust–Schiffrin technique do not show detectable surface plasmon resonance and CGNPs bigger than 100 nm may cause damage to the biological molecules, we have chosen to synthesize the CGNPs by the standard Turkevich method [27]. The size and density of the CGNPs can be determined from the absorbance peak and the corresponding wavelength [28]. This reaction also produces several by-products along with CGNPs, which can be removed by various techniques like centrifugation, ion exchange, dialysis, etc. [29]. Citrate gold nanoparticles (CGNP) are unstable in buffer solutions [30] and can be made stable by functionalizing them with polyethylene glycol (PEG) [31], which makes them withstand buffer solutions of molarity up to 1 M [32]. PEGylated gold nanoparticles (PGNP) are stable due to steric repulsions between the PEG chains [33]. NaOH can be used to replace the citrate capping on the gold nanoparticles with PEG terminated by thiol group (–SH) [34]. Thiol (–SH) group on the PEG ensures easy binding between gold nanoparticles and the PEG. The carboxyl (–COOH) group on the PEG can be activated with EDC/SNHS to make it suitable for conjugation with antibodies [35]. In [36], the authors presented different methods of conjugation of an antibody to the gold nanoparticle. One of these methods is the introduction of an antibody to the EDC/SHS-activated PGNP. Thus, antibodies corresponding to CD9/CD63/CD81 can be introduced to the activated PEG for isolating exosomes from serum.

In this study, we are reporting a gold nanoparticle-based fast and efficient immunoaffinity method in which PEGylated gold nanoparticles have been conjugated with anti-CD63 via ethyl (dimethyl aminopropyl) carbodiimide (EDC) and hydroxy sulfo succinimide (SNHS) chemistry. These antibody-conjugated gold nanoparticles are incubated with serum and centrifuged to isolate small exosomes (≤50 nm) in less than two hours. The isolation of exosomes is confirmed by characterization techniques such as TEM, DLS, NTA, and western blotting. 

## 2. Materials and Methods

### 2.1. Materials

The following chemicals were procured from Sigma Aldrich: gold (III) chloride hydrate (HAuCl4: Cat No. 254169), trisodium citrate dihydrate (Cat No. S1804), sodium hydroxide (NaOH—Cat No. S8045), poly(ethylene glycol) 2-mercaptoethanol ether acetic acid (PEG 3500 Da—Cat No. 757837), 2-(*N*- morpholino) ethanesulfonic acid (MES—Cat No. M5287), *N*- (3-Dimethylaminopropyl)-*N*-ethyl carbodiimide(EDC—Cat No. 39391), *N*-Hydroxysuccinimide (NHS—Cat No. 56480), *N*- Hydroxysulfosuccinimide sodium salt (SNHS-Cat No. 56485), phenylmethanesulfonyl fluoride(Cat No. P7626), bovine serum albumin (Cat No. 05470). Proteinase K was procured from MP Biomedicals LLC (Cat No. 193981). Amersham Hybond P 0.2µm polyvinylidene difluoride (PVDF) membrane (10600021) and Amersham ECL reagent (Cat No. RPN2235) were obtained from GE Health Care. Bradford reagent was obtained from Biorad (Cat No. 5000006). Non-fat milk powder and clot activator tubes (4 mL) were procured from local vendors. All other reagents and consumables were of reagent grade.

Primary antibodies: anti-CD63 (Cat No. PAB25155) was obtained from Abnova for western blotting. Anti-CD63 (Cat No. BS-1523R) was from ThermoFisher for functionalizing nanoparticle). Anti-CD9 (Cat No. A10789) and anti-CD81 (Cat No. A5270) were procured from ABclonal. Anti-albumin (Cat No. 1TT06396) was purchased from Immunotag. Anti-HSP70 was obtained from BD Biosciences (Cat No. 610608).

Secondary antibodies: Anti-rabbit (Cat No. ab131366) and anti-mouse (Cat No. ab131368) HRP were obtained from Abcam.

### 2.2. Methods

**Figure 1:**
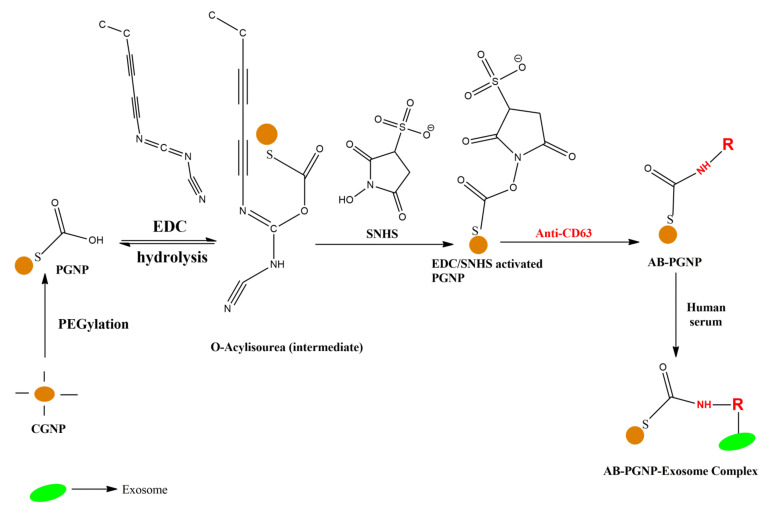
**A brief workflow of the conjugation of gold nanoparticles with exosomes**. The orange sphere represents the citrate-capped gold nanoparticle (CGNP). Upon incubation with PEG, it forms PEGylated gold nanoparticle (PGNP). Anti-CD63 antibody is conjugated to the PGNP after activating it with EDC/SNHS chemistry.

The workflow in this report is briefly presented in Figure 1. Freshly prepared citrate-capped gold nanoparticles (CGNPs) were PEGylated (i.e., incubated with PEG) to form PEGylated gold nanoparticles (PGNPs). These particles were functionalized with an antibody corresponding to the exosomal surface protein and finally incubated with human serum where the conjugation between CGNPs and exosomes took place. After centrifugation, PGNP-exosome complex settled at the bottom of the tube.

Stock solutions of Gold (III) Chloride Trihydrate and Trisodium Citrate Dihydrate were prepared at a molarity of 4.2 mM and 17 mM, respectively, and stored at 4 °C for future use. The prepared CGNPs were precipitated by centrifugation at 7000× *g* for 20 min at room temperature and dissolved in the required solvent (deionized water or sodium phosphate buffer (pH = 7.2, 25 mM). Upon optimization of CGNPs, bulk purification was conducted in 15 mL tubes in a centrifuge with parameter values the same as that in the micro centrifuge tubes. UV–Vis absorbance spectra of the GNP solutions were recorded for 1 mL of solution in a quartz cuvette in the absorbance range of 400 nm to 600 nm in a Perkin-Elmer Lambda 35 spectrophotometer.

#### 2.2.1. Synthesis of Gold Nanoparticles (CGNP’s)

Preparation at different temperatures took place as follows. A stock solution of 1 mL of gold (III) chloride trihydrate was mixed by magnetic stirrer with 18 mL of DI water. After complete mixing, the temperature of the hot plate was slowly increased in steps of 1 °C. At 80 °C, 1 mL of the trisodium citrate dihydrate (TCD) was added to the solution. The colour changed from golden yellow to red, indicating the formation of citrate-capped gold nanoparticles (CGNPs). At this point, the solution was allowed to reach room temperature and was then stored at 4 °C. The same procedure was repeated to synthesize CGNPs at different temperatures. 1 mL of CGNP solutions were pelleted, dissolved in DI water, and tested for UV–Vis absorbance.

Preparation at different TCD concentrations took place as follows. Experiments were performed to study the role of TCD in the synthesis of CGNPs. For this, 1 mL of gold (III) chloride trihydrate stock solution was mixed with 18.6 mL of DI water and heated at 80 °C, while simultaneously stirring. TCD (400 µL) stock solution was added when it became hot and, after some time, the solution turned from golden yellow to red. Finally, the solution was allowed to cool down to room temperature. A similar procedure was adopted to prepare three other solutions with different TCD stock solutions. The volume of the solution was always maintained to 20 mL by adjusting the amount of DI water to ensure uniformity in the measurements of the UV–Vis absorbance spectrum. Of these 4 CGNP solutions, 1 mL of each was tested by UV–Vis absorbance. 

Upon optimization of the parameters, such as temperature and TDS concentration, 140 mL of CGNPs was prepared in bulk.

#### 2.2.2. PEGylation of CGNPs Followed by the Activation of the Carboxyl Group of PEG for Conjugation with Antibody

PEGylated GNP (PGNP) solutions of 0.0033%, 0.0066% and 0.0099% were prepared by adding 1 mg, 2 mg, and 3 mg of PEG (3500 Da), respectively, in 30 mL of CGNP solution. This was followed by the addition of 1 M NaOH (to a final concentration of 24.39 mM) and incubated for 16 h at room temperature. 1 mL each of these solutions was pelleted and dissolved in DI water for checking UV–Vis absorbance. Similarly, 1 M NaCl was added (to a final concentration of 0.1 M) to the CGNP solution, which was incubated with PEG for 1 h at room temperature and pelleted. The pellet was dissolved in DI water for UV–Vis absorbance. The same procedure was repeated for incubation times of 18 h and 20 h. The optimized PGNPs could be stored at 4 °C for a period of one year.

Freshly prepared CGNPs, CGNPs after purification, and PGNPs were tested for zeta potential (Zetasizer Nano-ZS, Malvern Instruments) to analyse the effect of centrifugation on their stability. To activate the carboxyl group of PGNPs, freshly prepared 10% SNHS solution was used for all the experiments. Each parameter, i.e., pH of MES buffer, SNHS, and EDC, were optimized by performing experiments with one parameter being a variable while maintaining the other parameters at fixed values. Stock solutions of 1 M MES buffer with different pH values of 5.75, 6.17, and 6.48 were prepared in DI water. MES buffer was added to PGNPs for a final concentration of 24.39 mM, followed by the addition of EDC (for a final concentration of 0.877 mg/mL) and 100 µL of SNHS. This solution was incubated at room temperature for 30 min, pelleted, dissolved in DI water, and tested for UV–Vis absorbance. The same process was repeated by varying the pH values of the MES buffer.

Upon optimization of MES buffer, experiments were performed for the optimization of SNHS concentration. PGNPs in 24.39 mM MES buffer of pH 6.17 were placed in 1.5 mL microcentrifuge tube and then EDC was added for a final concentration of 0.877 mg/mL, followed by the addition of 20 µL of 10% SNHS solution, incubated for 30 min, pelleted by centrifugation, and dissolved in 1 mL of DI water. A similar process was repeated by varying the volume of SNHS and EDC, summarized in Table 2.

PGNPs were freshly activated, every time, for conjugating the antibody. After activation, they were pelleted and dissolved in 25 mM sodium phosphate buffer of pH 7.2 in order to make the buffer solution compatible with the antibody. 1 mL of activated PGNPs was incubated with 0.5 µL/mL of antibody with 1 mg/mL concentration. This sample was incubated for 2 h at 4 °C, after which it was pelleted at 4 °C and the whole supernatant was removed. The experiment was repeated with 0.75 µL/mL and 1 µL/mL of antibody. All these pellets were tested by western blotting to confirm the conjugation.

### 2.3. Western Blotting

The sample preparation involves treating the sample with SDS sample loading buffer and heating for 10 min at 97 °C. These samples were run in 12% SDS/PAGE, followed by blotting in PVDF membrane. The blot was blocked with 5% non-fat milk for 1 h at room temperature. The PVDF membrane with anti-CD63 conjugated PGNPs (AB-PGNPs) was probed with a corresponding secondary antibody at room temperature (27 °C) for 1 h. The PVDF membrane containing the exosomes prepared from the serum was probed with a primary antibody overnight at 4 °C, followed by a HRP conjugated secondary antibody. The blots were developed by using ECL reagents.

### 2.4. Serum Preparation

All the serum was prepared from blood collected from healthy volunteers. The RBCs of blood were allowed to clot in clot activator tubes, in an upright position, for 1 h at room temperature. This tube was then spun at 7000× *g* for 20 min. The serum, which is at the top of the tube, is collected using a pipette. Aliquots of 1 mL of serum were collected in 1.5 mL centrifugation tubes and stored at −20 °C for further experiments. For each experiment, 200 µL of serum was used.

### 2.5. Isolation of Exosomes from Serum

1 mL of anti-CD63 conjugated PGNPs (AB-PGNPs) and 200 µL of serum were incubated at 4 °C for 1 h. This solution was pelleted at 7000× *g* and the supernatant was completely removed. 50 µL of sodium phosphate buffer was then added to the pellet, heated at 37 °C for 1 min and pipetted to dissolve it completely. The concentrated exosomes were used for western blotting, DLS, NTA and TEM. 

### 2.6. Sample Preparation for TEM Analysis of GNPs

10 µL of CGNP solution was applied on a TEM grid and excess liquid was removed with a filter paper. This process was repeated twice. This was followed by three washes in DI water and allowed to air dry, following which the grid was viewed under a microscope.

### 2.7. Sample Preparation for TEM Analysis of Exosome-Conjugated GNP

Here, 1 mL of AB-PGNPs, conjugated with exosomes prepared from 200 µL of serum, was pelleted down and the pellet was washed in sodium phosphate buffer. After washing, it was dissolved in 50 µL of sodium phosphate buffer while heating the tube at 37 °C for about 3 min. The fully suspended exosome solution was mixed with 50 µL modified Karnovsky’s fixative and incubated for 1 h at 4 °C. The suspended exosomal solution was centrifuged at 4 °C for 20 min at 7000× *g* to form a pellet. The pellet formed after centrifugation was dissolved in 50 µL of sodium phosphate buffer at 37 °C. Subsequently, 10 µL of this solution was applied on a formvar/carbon-coated copper TEM grid and excess liquid was wicked off with filter paper. After this, the grid was washed with DI water for 1 min, followed by staining with 2% uranyl acetate at room temperature for 30 s. It was then washed thrice in DI water and allowed to air dry. The dried grid was ready for TEM viewing.

### 2.8. Transmission Electron Microscopy (TEM) and Exosome Size Distribution

All samples were drop mounted on formvar-carbon coated electron microscopy grids. The grids were observed under the JEOL 101 electron microscope (JEOL, Tokyo, Japan) at a magnification of 100 K. TEM micrographs were analysed using ImageJ. Linear ROIs were drawn to enumerate the size of the roughly spherical exosomes. Statistical analysis of the data was conducted to calculate the standard deviation and standard error of the mean. The sizes of the exosomes were then plotted as a scatter plot of different ranges against the mean size.

### 2.9. Sample Preparation for DLS, Zeta Potential, NTA and UV–Vis Absorbance

Zeta potential and DLS measurements were performed in Zetasizer Nano-ZS, Malvern Instruments. 1 mL each of CGNPs and PGNPs were used directly in a plastic cuvette for DLS and zeta potential. PGNP-exosome complex, obtained from 200 µL of serum, was made to 1 mL using sodium phosphate buffer for characterization.

NTA analysis was performed using NanoSight NS300, Malvern Panalytical Instruments. 1 mL of PGNPs was directly used, whereas PGNP-exosome complex obtained from 200 µL of serum was made to 1 mL using sodium phosphate buffer for characterization.

All the UV–Vis measurements were performed in Perkin-Elmer UV–Vis spectrophotometer with a sample volume of 1 mL.

### 2.10. Proteinase K Treatment of the Sample

1 mL of AB-PGNPs conjugated with 200 µL serum was pelleted down and the pellet was washed in sodium phosphate buffer. After washing the pellet, it was dissolved in 40 µL of sodium phosphate buffer. Proteinase K was added at a final concentration of 80 µg/mL and incubated for 45 min at room temperature. The proteinase K activity was stopped by adding 5 mM phenylmethanesulfonyl fluoride for 10 min at room temperature.

### 2.11. Total Protein Quantification

The exosome pellet dissolved in 50 µL sodium phosphate buffer was diluted 10 times. Next, 10 µL of diluted exosomes was added to 200 µL of Bradford Reagent in microtitre plates and incubated in the dark for 10 min. OD was taken at 595 nm. Bovine serum albumin was used as a reference standard sample.

### 2.12. Total RNA Isolation and Quantification

Here, 500 µL Trizol was added to the exosome pellet and vortexed to homogenise and incubated for 5 min at room temperature. To this, 200 µL of chloroform was added and shaken vigorously for 30 sec and incubated at room temperature for 10 min. The mixture was then centrifuged at 12,000× *g* for 15 min at 4 °C and the aqueous phase was collected. 500 µL of isopropanol was added to the collected aqueous phase, incubated at −80 °C for 1 h and centrifuged at 16,000× *g* for 15 min at 4 °C. The supernatant was discarded and the pellet was washed in 75% ethanol, centrifuged at 16,000× *g* for 15 min at 4 °C and dissolved in 20 µL RNase free water. A similar procedure was followed to quantify the RNA from cultured cells containing 2 × 10^5^ cells. RNA was quantified by means of nanodrop spectrophotometer. 

## 3. Results and Discussions

### 3.1. Characterization of CGNP & PGNP

**Figure 2:**
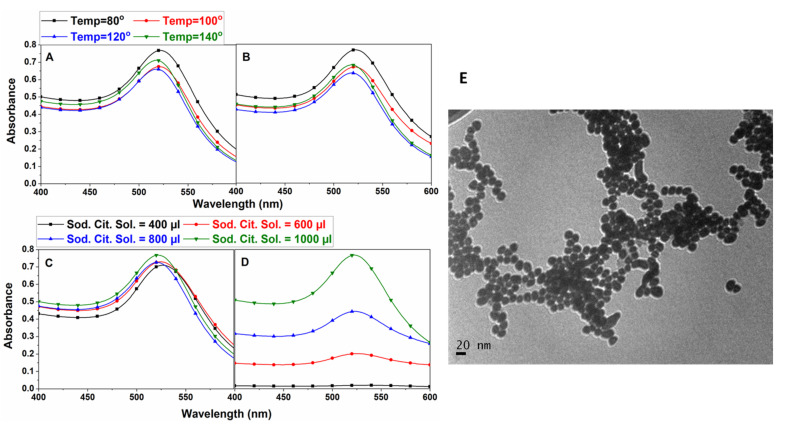
**UV absorbance curves of gold nanoparticles prepared at different temperatures and different volumes of sodium citrate stock solution**. (**A**) Temperature variations of GNP before purification; (**B**) temperature variations of GNP after purification; (**C**) sodium citrate variation before purification; (**D**) sodium citrate variation after purification; (**E**) TEM characterization of GNP after purification. The TEM characterization was performed 3 times and the UV–Vis characterization was performed several times.

Gold nanoparticles, as mentioned, were synthesized by the standard Turkevich method [27]. Optimization was conducted by varying gold (III) chloride Trihydrate and TCD individually. Particles of different sizes were synthesized at different temperatures (80 °C, 100 °C, 120 °C, and 140 °C), with absorption peaks ranging from 515 nm to 525 nm (Figure 2A). Purification of these particles was conducted at 7000× *g* by removing most of the supernatant, but this also reduced the max absorbance peak (by 1% to 5%), except for the CGNPs synthesized at 80 °C. Similarly, experiments were performed to study the role of TCD at different concentrations in the synthesis of CGNPs (absorption spectra in Figure 2C,D), which show a reduction in the stability of CGNPs with TCD concentration. Hence, 1 mL of TCD stock solution was used for preparing 20 mL of CGNPs for future experiments.

Upon optimization of gold (III) chloride trihydrate and TCD concentrations, bulk preparation and purification of CGNPs was conducted. The concentration of CGNPs were calculated theoretically as 7.4 × $10^{8}$/mL, using the procedure from [28]. The surface potential of freshly prepared CGNPs before and after purification were found to be −33.26 mV and −15.7 mV, respectively, by measurement of zeta potential. The reduction in the magnitude of zeta potential upon purification shows the removal of the citrate capping from the surface of the GNPs due to centrifugation. To avoid excessive increase in the concentration of CGNPs that causes their reduced interaction with biomolecules, pellets corresponding to 20 mL of CGNPs dissolved in 10 mL of DI water were used for all further experiments.

**Figure 3:**
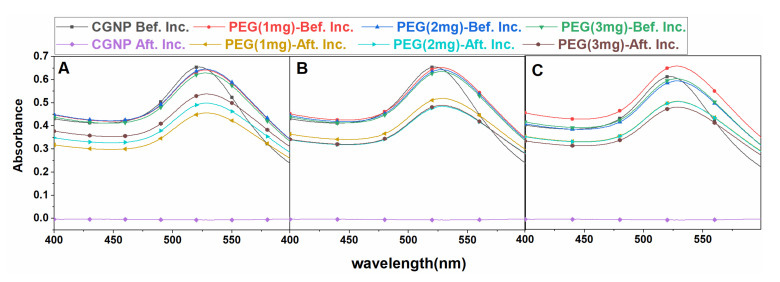
UV–Vis absorbance curves of PGNP by incubation of CGNP with different concentrations of PEG before and after incubation with 0.1 M NaCl at (**A**) 16 h, (**B**) 18 h, (**C**) and 20 h. **The experiment was performed several times**.

The TEM picture shows the size of CGNPs prepared at 80 °C are approximately 20 nm (Figure 2F). Introducing CGNPs to 0.1 M NaCl immediately led to a reduction in the absorbance peak (Figure 3), which shows their inability to be used in buffer solutions. Hence, polyethylene glycol (PEG) was used to functionalize CGNPs.

PEGylation of CGNPs, i.e., adhesion of PEG to the CGNPs, is based on the availability of a bare surface on the CGNPs. The already existing citrate capping prevents the interaction of PEG with the CGNP’s surface, which can be partially solved by the addition of NaOH, which leads to the removal of citrate capping on the CGNPs, thereby creating a more bare surface for interaction with PEG on the CGNPs. On the other hand, the addition of NaOH leads to the immediate aggregation of CGNPs that are highly unstable, which can be clearly seen by the visible colour change (from red to purple) of the CGNP solution, which indicates an increase in the particle size. The same was confirmed by the shift in the UV–Vis absorbance peaks of PGNPs (Figure 3A) by 10 nm (i.e., at 530 nm), as compared to that of CGNPs (Figure 2B). The time also plays a significant role in PEGylation when 16 h, 18 h, and 20 h reactions were compared, as the 20 h incubation time of CGNPs showed better stability in 0.1 M NaCl solution (Figure 3). Therefore, PEGylation was performed for 20 h with 0.0033% PEG for the rest of the experiments. The surface potential of this sample was −36.08 mV, which is greater compared to that of CGNPs before and after purification. Thus, PEGylation enhanced the stability of the nanoparticles even after multiple centrifugations thoroughout the process. The particle count of PGNPs was $3.1\times 10^{7}$/mL with an approximate size of 28.5 nm.

### 3.2. Antibody Conjugation to the PGNP and Exosome Isolation

**Figure 4:**
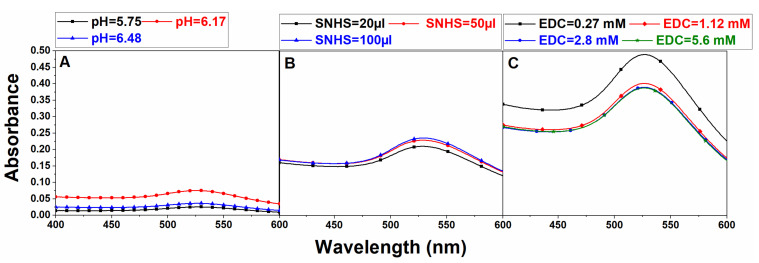
**UV–Vis absorbance curves corresponding to the optimization of –COOH of PEG conjugated with GNP**. (**A**) Experiments with different variations of MES buffer pH; (**B**) experiments with different variations of SNHS solution; (**C**) experiments with different variations of EDC. The experiments were performed several times.

Carboxyl groups (–COOH), upon reaction with EDC, produce O-acylisourea, which is unstable in nature and, during hydrolysis, converts back to –COOH. Hence, the PGNPs with the terminal carboxyl (–COOH) were activated with EDC/NHS or EDC/SNHS to make them suitable for conjugation with the antibody. Experiments were performed to optimize the parameters varying the concentrations of pH of MES buffer, SNHS, and EDC, successively. Theoretically, the cross-linking of EDC/SNHS with COOH should be optimal for low values of pH. However, in our experiments, a noticeable pellet after centrifugation was formed only for a pH value of 6.17, clearly evident from the absorption spectra (Figure 4A). Hence, a pH of 6.17 was used for further experiments. Similarly, the concentration of SNHS was optimized based on the maximum absorption, which is shown in Figure 4B. A series of experiments were conducted for optimizing the concentrations of EDC. A higher absorption peak was seen for the sample with the lowest amount of EDC concentration and, hence, 0.0438 mg/mL of EDC was chosen for further experiments.

**Figure 5:**
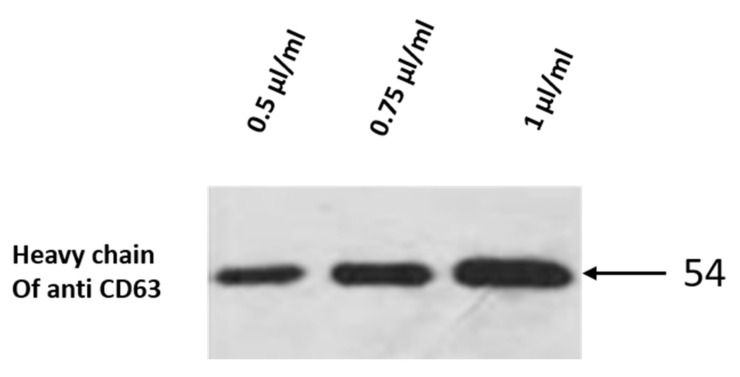
**Western blot performed on gold nanoparticle conjugated with anti-CD63**. The blot shows the western results of SDS-PAGE loaded with GNP conjugated with different concentrations of anti-CD63. The blot is incubated with secondary antibody and developed. The experiment was performed several times.

COOH-activated PGNPs were incubated with different concentrations of anti-CD63 for 2 h and the conjugation of PGNPs was confirmed by western blotting. The developed image of the blot is presented in Figure 5. The band at about 50 KDa corresponds to the heavy chain of anti-CD63 antibody that is conjugated with the PGNPs. Since 1 µL /mL antibody produces a better band, PGNPs were incubated, for all future experiments, with 1 µL/mL of antibody. Increasing the concentration of antibody beyond this or increasing the incubation time beyond 2 h led to the complete aggregation of PGNPs. Such samples showed flat UV–Vis absorbance curves without any peak. Finally, the anti-CD63 antibody-conjugated PGNPs were incubated with human serum for 1 h to form the PGNP-exosome complex. Pellets were formed at different speeds and it was noticed that the pellet density was small for the sample that was spun at 5000× *g*, as smaller CGNPs will not settle at lesser centrifugation speeds. Samples pelleted at 7000× *g* and 9000× *g* had an approximately similar density. However, usage of high g-force might lead to the rupture of the exosome. Hence, a centrifugation speed of 7000× *g* was used for pelleting the sample. 

Using a Bradford assay, the concentration of protein was found to be 2 µg/µL. The total protein yield from 200 µL serum was found to be 100 µg. The RNA was found to be 45 ng/µL. The total RNA yield from 200 µL serum was found to be 900 ng. The concentration of RNA from cells measured as reference was found to be 345 ng/µL and the total yield from 2 × 10^5^ cells 6.9 µg.

**Figure 6:**
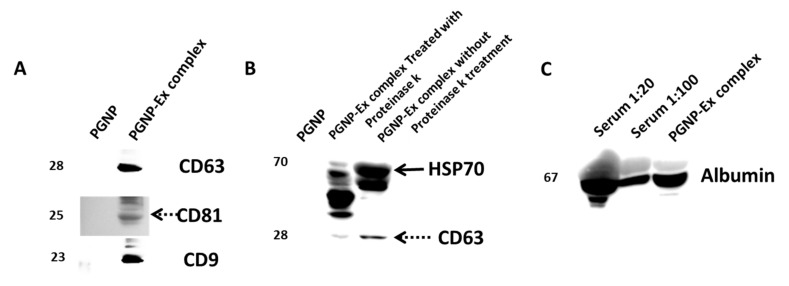
**Western blot results of SDS-PAGE loaded with PEGylated gold nanoparticle conjugated with exosomes**. (**A**) Blot showing exosomal surface proteins CD9, CD63, CD81 at weights 23, 28 and 25 kDa respectively. (**B**) Blot showing HSP70 and CD63 from the samples with and without proteinase k treatment. (**C**) Blot showing reduced presence of albumin in the exosome sample. 40 µL volumes were used for all the samples. To prepare 1:20 dilution of serum, 2 µL serum was mixed with 38 µL sodium phosphate buffer. A 1:100 dilution of serum was prepared by adding 1 µL of serum to 99 µL of sodium phosphate buffer. A 1 mL antibody-conjugated PGNP was incubated with 200 µL serum and pelleted using centrifugation. The PGNP-exosome pellet was dissolved in 40 µL of sodium phosphate buffer and loaded. Western blotting for albumin was performed twice and the blots for HSP70, CD9, CD63, and CD81 were repeated thrice.

In order to test the presence of different categories of proteins, such as transmembrane proteins, cytosolic proteins, and extracellular proteins, several western blotting experiments were planned. Samples of the PGNP-exosome complex were prepared and used for electrophoresis, followed by western blotting. The samples were probed with antibodies against CD9, CD63, CD81, HSP70 and albumin, followed by a suitable secondary antibody. The developed images of the blots are presented in Figure 6. 

The reduction in the band intensity of HSP-70 in the sample treated with proteinase k may be due to the digestion of proteins outside the exosomal membrane (Figure 6B). However, it shows that the HSP-70 inside the membrane remains, which ensures the quality of our preparation. The exosome sample was also probed with anti-human albumin (Figure 6C). Since 200 µL serum was used to prepare the exosome and compared with 2 µL of serum and 0.4 µL of serum (diluted sample), we can conclude that the serum albumin has reduced manifold in the purified exosome sample. Hence, it can be assumed that very minimal albumin contamination exists in the isolated exosomes. Similar observations have been reported by a different group [37].

**Figure 7:**
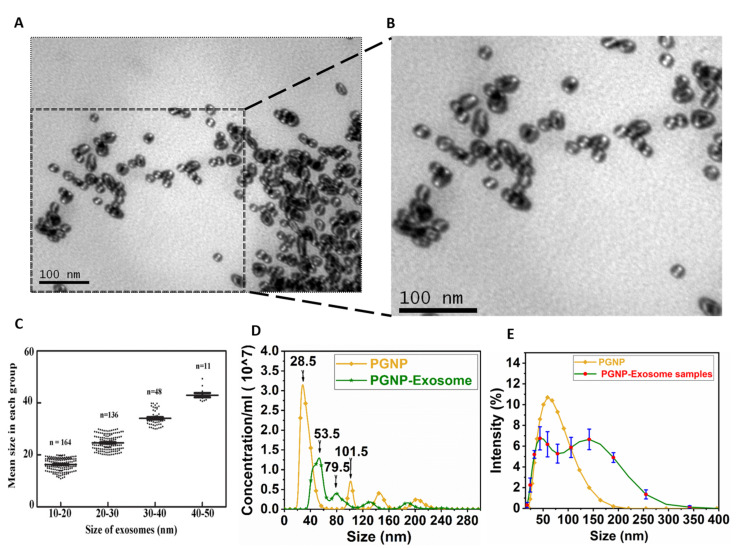
(**A**) **Characterization of exosomes**. The pellet formed after centrifugation of PGNP conjugated with exosome is washed with sodium phosphate buffer. It is then incubated in modified Karnovskys fixative, centrifuged to remove the supernatant, and dissolved back in the sodium phosphate buffer. TEM grid is prepared by using this solution followed by staining with uranyl acetate solution. (**B**) **The box in A is shown as zoomed**. (**C**) **Grouped scatter plot showing the size distribution of the exosome population obtained**. Standard error of mean (SEM) is shown on the scatter plot. (**D**) **Size distribution and quantification of PGNP-exosome and exosomes using NTA**. (**E**) **Size distribution of PGNP and PGNP-exosome complex using DLS**. **The curve is a mean curve of four sets of data. SEM of four experiments is shown**. TEM characterization and DLS was performed 4 times and NTA was conducted once.

Figure 7A shows the presence of membranous structures seen by transmission electron microscopy. The TEM images show that the nanovesicles captured are within the size range corresponding to that of exosomes. The smaller vesicles appear membranous and similar in morphology to the larger vesicles under the TEM. Such vesicles have earlier been reported to be obtained in the asymmetrical flow field-flow fractionation method of exosome characterization [3]. Figure 7B shows a zoomed-in portion of Figure 7A. The difference between the structures of exosomes and PGNP is very evident from Figure 7A and Figure 2E. PGNPs appear to be spherical and dense, which is in contrast to that of exosomes, which are irregular in shape and membrane structured. Upon manually evaluating the particles using the TEM image, we plotted a curve (Figure 7C). It was understood that nearly 77% of the vesicles obtained were in the reported size range for exosomes.

Figure 7D shows the NTA results of PGNP and PGNP-exosome complex. The PGNP concentration is $3.25\times 10^{7}$/mL with an average particle size of 28.5 nm, the concentration of PGNP-exosome complex is $1.25\times 10^{7}$/mL and the maximum number of particles were found at 53.5 nm (NTA). From this particle count, it can be seen that nearly 41% of PGNPs were conjugated with the exosomes. 

Figure 7E shows the DLS results of PGNP and PGNP-exosome complex. Results show a significant portion of the particles corresponding to sizes ranging from 25 nm to 200 nm. Similar results were obtained for the samples stored at −20 °C for two days, which show the stability of isolated exosomes for at least two days. The results of NTA and DLS have some similarities. However, some differences exist between the results of TEM and DLS. In [38,39], the authors presented the reasons for the deviation in the sizes of extracellular vesicles measured using TEM, NTA, and DLS. TEM measures the inhomogeneity in the electron densities of the samples. Due to a significant difference in the electron densities between the core and the capping region of the extracellular vesicles, TEM provides the size of the extracellular vesicle alone. However, in NTA and DLS, the size is measured by considering the diffusion coefficient of the particles in the solution. Since the diffusion coefficient of the extra cellular vesicles depends on their capping density (which includes antibody, PEG, gold nanoparticles), the size appears to be bigger than that of the measurement taken by TEM.

The efficiency of the PGNP to pull down the exosome could depend on several factors. The first factor may be the PEG molecule used for PEGylation of CGNP. Since the shape of the PEG molecule is irregular in nature, it could block a significant surface area of the CGNP during PEGylation. This impact may be directly proportional to the molecular weight of PEG. As the molecular weight of the PEG increases, the surface area occupied on the CGNP increases. So the probability of more PEG molecule binding to the CGNP during PEGylation reduces. This reduces the total number of COOH groups that are necessary for conjugation with the anti-CD63 antibody. Hence, using PEG of a lower molecular weight could be a better option. The second factor may be the size of the CGNP. If the size of the CGNP is larger, then the number of PEG molecules binding to its surface increases. This directly increases its binding ability with exosomes. Further, since the weight of the CGNP increases with its size, the g force at which it should be spun can be reduced. Apart from these two major factors, the concentration of PGNP can also impact the isolation of exosomes. We also assume that increasing the size of the CGNP helps in pulling down exosomes of bigger size. Optimizing all these factors is beyond the scope of this work.

Due to the presence of the conventional surface proteins (CD9, CD63, CD81) of exosomes and since the size range of the isolated particles is within the accepted exosomal range, we prefer to name them as small exosomes.

## 4. Conclusions

Exosomes of various sizes have recently gained attention due to their involvement in several diseases, RNA exchange, and immune responses. Here, detailed investigations have been conducted for optimizing the predominant isolation of small exosomes from blood-derived serum. In this technique, gold nanoparticles were conjugated with CD63 antibody by using a PEG functionalized with Thiol (–SH) and carboxyl (–COOH) groups at its terminals, and this conjugation was confirmed by western blotting. AB-PGNPs were further incubated with serum for conjugating with exosomes. The isolation of small exosomes (≤50 nm) was confirmed by western blotting, DLS, TEM, and NTA. Based on these techniques, it can be concluded that by adding additional weight to exosomes using PGNPs, they can be separated from serum using a bench top centrifuge at a low g-force of 7000× *g*. Serum incubation and separation of exosomes requires less than two hours of time if antibody-conjugated gold nanoparticles are available. The isolated small exosomes were found to be stable for more than two days, when stored at −20 °C. This method can be used for separating the small exosomes from any biofluid without any need for pre-enrichment of the sample. 

We believe that the proposed technique will be very valuable in advanced studies of exosomal subcategories and this technique can also be extended to differently sized sEVs.

## Figures and Tables

**Table 1:** Current Methods of Exosome Isolation and their advantages and disadvantages.

Procedure.	Equipment	Advantages	Disadvantages/Comments	Size Range of Exosomes Isolated (In Nano Meter)
**Ultra Centrifugation** [2,10]	**Ultracentrifuge**	**High sample capacity**	**Dependency on expensive instrument,** **Low purity,** **Time-consuming**	**20–150**
**Density Gradient Centrifugation** [11,12]	**Ultracentrifuge**	**High separation efficiency,** **High purity,** **Low probability of particles getting crushed/deformed due to high speed**	**Dependency on expensive instrument,** **Long run time,** **Significant space consumption in laboratory**	**30–120**
**Chromatography** [17,18]	**Gel Filtration Column**	**High purity,** **Uniform size**	**Need for more laboratory equipment,** **Low processing volume**	**50–150**
**Immunomagnetic Beads** [19]	**Magnetic Beads**	**Easily available,** **No need for expensive instruments**	**Low processing volume,** **Long incubation time**	**30–300**
**Commercial Kits** [12]	**Bench-top centrifuge**	**Easy to handle,** **Fast isolation**	**High reagent cost**	**80–140**
**Our Preparation**	**Bench-top centrifuge**	**Easy to handle** **Fast isolation**	**Reagent cost (excluding instrument and other costs) approximately USD 2.5 /200 µL serum**	**≤50**

**Table 2:** Optimization of carboxyl group of PEG conjugated with GNP.

Step no.	Fixed Parameters	Variable Parameter	Optimized Value
**1**	EDC = 0.877 mg/mL; SNHS = 100 µL/mL	pH = 5.75	6.17
		pH = 6.17	
		pH = 6.48	
**2**	pH = 6.17; EDC = 0.877 mg/mL	SNHS (µL/mL) = 20	100 µL
		SNHS (µL/mL) = 50	
		SNHS (µL/mL) = 100	
**3**	pH = 6.17; SNHS = 100 µL/mL	EDC (mg/mL)= 0.0438	0.0438 mg/mL
		EDC (mg/mL)= 0.17	
		EDC (mg/mL)= 0.43	
		EDC (mg/mL)= 0.877	

## Data Availability

Data sharing not applicable.

## References

[1] Bobrie A., Colombo M., Krumeich S., Raposo G., Théry C. (2012). Diverse Subpopulations of Vesicles Secreted by Different Intracellular Mechanisms Are Present in Exosome Preparations Obtained by Differential Ultracentrifugation. J. Extracell. Vesicles.

[2] Lamparski H.G., Metha-Damani A., Yao J.Y., Patel S., Hsu D.H., Ruegg C., Le Pecq J.B. (2002). Production and Characterization of Clinical Grade Exosomes Derived from Dendritic Cells. J. Immunol. Methods.

[3] Sitar S., Kejžar A., Pahovnik D., Kogej K., Tušek-Žnidarič M., Lenassi M., Žagar E. (2015). Size Characterization and Quantification of Exosomes by Asymmetrical-Flow Field-Flow Fractionation. Anal. Chem..

[4] Filipazzi P., Bürdek M., Villa A., Rivoltini L., Huber V. (2012). Recent Advances on the Role of Tumor Exosomes in Immunosuppression and Disease Progression. Seminars in Cancer Biology.

[5] Vlassov A.V., Magdaleno S., Setterquist R., Conrad R. (2012). Exosomes: Current Knowledge of Their Composition, Biological Functions, and Diagnostic and Therapeutic Potentials. Biochimica et Biophysica Acta—General Subjects.

[6] Wendler F., Bota-Rabassedas N., Franch-Marro X. (2013). Cancer Becomes Wasteful: Emerging Roles of Exosomes in Cell-Fate Determination. J. Extracell. Vesicles.

[7] Li W., Li C., Zhou T., Liu X., Liu X., Li X., Chen D. (2017). Role of Exosomal Proteins in Cancer Diagnosis. Mol. Cancer.

[8] Zhang H.G., Zhuang X., Sun D., Liu Y., Xiang X., Grizzle W.E. (2012). Exosomes and Immune Surveillance of Neoplastic Lesions: A Review. Biotech. Histochem..

[9] Isola A., Chen S. (2016). Exosomes: The Messengers of Health and Disease. Curr. Neuropharmacol..

[10] Manna I., de Benedittis S., Quattrone A., Maisano D., Iaccino E., Quattrone A. (2020). Exosomal MiRNAs as Potential Diagnostic Biomarkers in Alzheimer’s Disease. Pharmaceuticals.

[11] KW P., Kierulf B. (2015). Direct Isolation of Exosomes from Cell Culture: Simplifying Methods for Exosome Enrichment and Analysis. Transl. Biomed..

[12] Helwa I., Cai J., Drewry M.D., Zimmerman A., Dinkins M.B., Khaled M.L., Seremwe M., Dismuke W.M., Bieberich E., Stamer W.D. (2017). A Comparative Study of Serum Exosome Isolation Using Differential Ultracentrifugation and Three Commercial Reagents. PLoS ONE.

[13] Théry C., Amigorena S., Raposo G., Clayton A. (2006). Isolation and Characterization of Exosomes from Cell Culture Supernatants and Biological Fluids. Curr. Protoc. Cell Biol..

[14] Yu L.L., Zhu J., Liu J.X., Jiang F., Ni W.K., Qu L.S., Ni R.Z., Lu C.H., Xiao M.B. (2018). A Comparison of Traditional and Novel Methods for the Separation of Exosomes from Human Samples. Biomed. Res. Int..

[15] Whiteside T.L. (2015). The Potential of Tumor-Derived Exosomes for Noninvasive Cancer Monitoring. Expert Rev. Mol. Diagn..

[16] Yuana Y., Levels J., Grootemaat A., Sturk A., Nieuwland R. (2014). Co-Isolation of Extracellular Vesicles and High-Density Lipoproteins Using Density Gradient Ultracentrifugation. J. Extracell. Vesicles.

[17] Szatanek R., Baran J., Siedlar M., Baj-krzyworzeka M. (2015). Isolation of Extracellular Vesicles: Determining the Correct Approach (Review). Int. J. Mol. Med..

[18] Benedikter B.J., Bouwman F.G., Vajen T., Heinzmann A.C.A., Grauls G., Mariman E.C., Wouters E.F.M., Savelkoul P.H., Lopez-Iglesias C., Koenen R.R. (2017). Ultrafiltration Combined with Size Exclusion Chromatography Efficiently Isolates Extracellular Vesicles from Cell Culture Media for Compositional and Functional Studies. Sci. Rep..

[19] Baranyai T., Herczeg K., Onódi Z., Voszka I., Módos K., Marton N., Nagy G., Mäger I., Wood M.J., El Andaloussi S. (2015). Isolation of Exosomes from Blood Plasma: Qualitative and Quantitative Comparison of Ultracentrifugation and Size Exclusion Chromatography Methods. PLoS ONE.

[20] Martín-Gracia B., Martín-Barreiro A., Cuestas-Ayllón C., Grazú V., Line A., Llorente A., De La Fuente J.M., Moros M. (2020). Nanoparticle-Based Biosensors for Detection of Extracellular Vesicles in Liquid Biopsies. J. Mater. Chem. B.

[21] Gao Y., Qin Y., Wan C., Sun Y., Meng J., Huang J., Hu Y., Jin H., Yang K. (2021). Small Extracellular Vesicles: A Novel Avenue for Cancer Management. Front. Oncol..

[22] Zhang H., Freitas D., Kim H.S., Fabijanic K., Li Z., Chen H., Mark M.T., Molina H., Martin A.B., Bojmar L. (2018). Identification of Distinct Nanoparticles and Subsets of Extracellular Vesicles by Asymmetric Flow Field-Flow Fractionation. Nat. Cell Biol..

[23] Liangsupree T., Multia E., Riekkola M.L. (2021). Modern Isolation and Separation Techniques for Extracellular Vesicles. J. Chromatogr. A.

[24] Lee S.S., Won J.H., Lim G.J., Han J., Lee J.Y., Cho K.O., Bae Y.K. (2019). A Novel Population of Extracellular Vesicles Smaller than Exosomes Promotes Cell Proliferation. Cell Commun. Signal..

[25] Zhang Q., Jeppesen D.K., Higginbotham J.N., Graves-Deal R., Trinh V.Q., Ramirez M.A., Sohn Y., Neininger A.C., Taneja N., McKinley E.T. (2021). Supermeres Are Functional Extracellular Nanoparticles Replete with Disease Biomarkers and Therapeutic Targets. Nat. Cell Biol..

[26] Zhao P., Li N., Astruc D. (2013). State of the Art in Gold Nanoparticle Synthesis. Coord. Chem. Rev..

[27] Turkevich J., Stevenson P.C., Hillier J. (1951). A Study of the Nucleation and Growth Processes in the Synthesis of Colloidal Gold. Discuss. Faraday Soc..

[28] Haiss W., Thanh N.T.K., Aveyard J., Fernig D.G. (2007). Determination of Size and Concentration of Gold Nanoparticles from UV-Vis Spectra. Anal. Chem..

[29] Balasubramanian S.K., Yang L., Yung L.Y.L., Ong C.N., Ong W.Y., Yu L.E. (2010). Characterization, Purification, and Stability of Gold Nanoparticles. Biomaterials.

[30] Baptista P., Doria G., Henriques D., Pereira E., Franco R. (2005). Colorimetric Detection of Eukaryotic Gene Expression with DNA-Derivatized Gold Nanoparticles. J. Biotechnol..

[31] Harrison E., Hamilton J.W.J., Macias-Montero M., Dixon D. (2017). Peptide Functionalized Gold Nanoparticles: The Influence of PH on Binding Efficiency. Nanotechnology.

[32] Manson J., Kumar D., Meenan B.J., Dixon D. (2011). Polyethylene Glycol Functionalized Gold Nanoparticles: The Influence of Capping Density on Stability in Various Media. Gold Bull..

[33] Stiufiuc R., Iacovita C., Nicoara R., Stiufiuc G., Florea A., Achim M., Lucaciu C.M. (2013). One-Step Synthesis of PEGylated Gold Nanoparticles with Tunable Surface Charge. J. Nanomater..

[34] Conde J., Baptista P.V., Hernández Y., Sanz V., De La Fuente J.M. (2012). Modification of Plasmid DNA Topology by Histone-Mimetic Gold Nanoparticles. Nanomedicine.

[35] Vashist S.K. (2012). Comparison of 1-Ethyl-3-(3-Dimethylaminopropyl) Carbodiimide Based Strategies to Crosslink Antibodies on Amine-Functionalized Platforms for Immunodiagnostic Applications. Diagnostics.

[36] Jazayeri M.H., Amani H., Pourfatollah A.A., Pazoki-Toroudi H., Sedighimoghaddam B. (2016). Various Methods of Gold Nanoparticles (GNPs) Conjugation to Antibodies. Sens. Bio-Sens. Res..

[37] Lötvall J., Hill A.F., Hochberg F., Buzás E.I., Vizio D.D., Gardiner C., Gho Y.S., Kurochkin I.V., Mathivanan S., Quesenberry P. (2014). Minimal Experimental Requirements for Definition of Extracellular Vesicles and Their Functions: A Position Statement from the International Society for Extracellular Vesicles. J. Extracell. Vesicles.

[38] Varga Z., Fehér B., Kitka D., Wacha A., Bóta A., Berényi S., Pipich V., Fraikin J.L. (2020). Size Measurement of Extracellular Vesicles and Synthetic Liposomes: The Impact of the Hydration Shell and the Protein Corona. Colloids Surf. B Biointerfaces.

[39] Lyu T.S., Ahn Y., Im Y.J., Kim S.S., Lee K.H., Kim J., Choi Y., Lee D., Kang E.S., Jin G. (2021). The Characterization of Exosomes from Fibrosarcoma Cell and the Useful Usage of Dynamic Light Scattering (DLS) for Their Evaluation. PLoS ONE.

